# Barriers to Family Planning Service Utilization in Ethiopia: A Qualitative Study

**DOI:** 10.4314/ejhs.v33i2.8S

**Published:** 2023-10

**Authors:** Meskerem Jisso, Netsanet Abera Assefa, Akalewold Alemayehu, Anteneh Gadisa, Rekiku Fikre, Abdurezak Umer, Hussen Mohammed, Bekele Yazie, Habtamu Sime Gizaw, Biru Abdissa Mizana, Elias Ali Yesuf, Binyamm Tilahun, Berhanu Fikadie Endehabtu, Tajebew Zayede Gonete, Kassahun Dessie Gashu, Dessie Abebew Angaw, Kassu Ketema Gurmu, Alemu Tamiso

**Affiliations:** 1 Hawassa University, College of Medicine and Health Sciences, Ethiopia; 2 Dire Dawa University, College of Medicine and Health Sciences, Ethiopia; 3 University of Gonder, College of Medicine and Health Science, Institute of Public Health, Ethiopia; 4 Jimma University, Institute of Health, Jimma, Ethiopia; 5 World Health Organization Country Office for Ethiopia, Universal Health Coverage/Life Course, Health System Strengthening Team, Addis Ababa, Ethiopia

**Keywords:** Barriers, Ethiopia, Family Planning, Misconception

## Abstract

**Background:**

The unmet need for family planning (FP) is a major impediment to achieving the sustainable development goal The COVID-19 pandemic and other contextual, individual, and hospital-related problems are major barriers that reduce FP service uptake. However, most of the studies are quantitative and give due focus to individual and community-level barriers. Therefore, this study tends to explore barriers to the utilization of FP in Ethiopia including health care and contextual barriers.

**Methods:**

A multiple explorative case study design was employed from October to December 2021 and a total of 41 Key-informant interviews, 32 in-depth interviews, and 13 focus group discussions were performed by using the purposive sampling technique. The data were analyzed with a thematic content analysis approach using NVivo software.

**Result:**

This study explored barriers to FP in four major teams; individual, community-related, health system, and contextual barriers. It reviled that the community's misconception, fear of side effects, lack of women's decision-making autonomy, existing socio-cultural norms, religious conditions, topography, covid 19 pandemic, and conflict were the major barriers to FP service utilization.

**Conclusion:**

Using the four teams mentioned above, this study identified different poor health professional skills, misconceptions, pandemics, functional, and structurally related barriers. As a result, it is recommended that health education for the community and training for health professionals are important. Collaboration between government and non-government organizations is also mandatory for strengthening mentorship and supervision systems and establishing resilient health care that can avoid future pandemics.

## Introduction

Family planning (FP) plays an important role in the lives of individuals, communities, and countries (1). It aids in the prevention of unwanted pregnancies, sexually transmitted infections (STIs), maternal and child health, and educational possibilities for women. Furthermore, it decreases demographic transition, which reduces competition and resource dilution, thereby boosting the country's economy (2).

Even though FP is one of the most important indicators for assessing universal access to sexual and reproductive health care services to achieve the Sustainable Development Goals, particularly target 3.7, the burden of unmet FP needs is a significant obstacle to achieving the goal. About 190 million reproductive-age women have an unmet need for contraception, which worsens in developing countries, where about 20% of women could not avoid pregnancy even if they wanted to (3).

Over the last few decades, Ethiopia has made significant progress toward obtaining universal health care, notably in terms of access to and utilization of family planning services. Although the use of modern contraceptive methods has increased from 14% in 2005 to 41% in 2019, the unmet need for FP remains a significant barrier; 22% of reproductive-age women have an unmet need for FP (4,5).

On the other hand, family planning services have been interrupted by the COVID-19 pandemic. During the pandemic, an estimated 12 million women were unable to access contraception. Modeling of the coronavirus pandemic's indirect supply- and demand-side effects on maternal mortality in low- and middle-income countries, including Ethiopia, has revealed that even modest service reduction scenarios can result in a substantial number of unwanted pregnancies (6). Globally, due to covid-19 disruption about 4.6 million women are exposed to unintended pregnancy and the problem is also in among developing countries like Ethiopia.

In addition to Covid 19 Effect, different studies stated that myths and misconceptions, known side effects, health provider-related barriers, restrictive cultural norms, and inaccessibility to health care services are among the underlying causes of this low service utilization (7–9). However, most of the studies are quantitative and focus on individual and community-level barriers. As a result, the goal of this study is to explore health care and contextual barriers to the utilization of FP in Ethiopia.

## Materials and Methods

**Study area and design:** The research was conducted between October and December 2021 in four Ethiopian regions: Oromia, Sidama, Amhara, and the South Nation Nationality of People (SNNP), and it included a variety of geographic and agro-economic situations, including urban, rural, and equity zones.

**Study design:** We deployed a multiple explorative case study design, where the primary health care unit (PHCU), which includes primary hospitals, health centers, and health posts, was assigned as a “case”. Multiple exploratory cases were included in the study to compare the phenomenon of interest within and across cases and contexts to make the findings more compelling. It involves key-informant interviews, and in-depth interviews, and focuses on group discussion within a multiple explorative case study design.

**Participants:** Community and health care representatives were involved in the study. Community representatives including women of reproductive age groups, community leaders, women and youth group members, traditional healers, traditional birth attendants, and health development teams were included in the study. The quality improvement team, facility maternal and child health (MCH) focal, and RMNH experts at regional, zonal, and district levels were included in the qualitative study.

**Sample size and sampling:** In conjunction with regional health bureaus, FGD, KII, and IDI participants were selected from all quarters of the health system including the community. For the key-informant and in-depth interview, participants were selected using a maximum variation sampling. In maximum variation sampling, participants' selection is often made using pre-set criteria to ensure the inclusion of as many variant observations as possible. These variations can result from variations in the demography of the participants or the phenomenon. In this study, maximum variations were achieved by including participants from different geographical locations, ages, gender, and role in the community and health care. FGD participants were purposively selected in the community. The sample size was determined by the level of saturation and redundancy of the collected information.

A total of 41 participants (regional RMNCH directors, Woreda RMNCH coordinators, PHCU MCH case team leaders) participated in the key informant interviews (KIIs), whereas 32 people (pregnant mothers, mothers who gave birth in the facility and in at their home, and both male and female youths) participated in the in-depth interview (IDI). A total of 13 focus group discussions (FGDs) were undertaken considering agrarian, urban, and equity contexts. Participants in the focus group included community leaders, health development team members, religious leaders, traditional healers, and traditional birth attendants. Because of the cultural sensitivity of the SRMH services, clients who participated in IDI have not participated in the FGD.

**Tools and procedures:** A pre-tested key-informant, in-depth interview, and focus group discussion guides were used for collecting relevant data. The tools were developed to find out conditions that facilitate or impede FP service utilization by individuals and demand-side barriers. The tools were as flexible as possible to capture wider factors that influence the utilization of FP services.

**Data quality Assurance:** Training has been provided to data collectors and supervisors about the purpose and content of the tools prior to the data collection. A pre-test was also undertaken prior to the actual data collection in all regions' Woredas other than the actual data collection area. Close follow-up by the research team and supervisors was undertaken and feedback was also given on the completed forms for the data collectors before the next data collection day. A quality check was done after transcription, to check the consistency of the transcription with the recorded audio and way of writing.

**Data management and analysis:** For analysis, the data were transcribed and then translated into English. The quality of the verbatim transcriptions and translations was assessed by a team of experts. To clarify issues, field notes were added to the transcripts. Researchers read the transcripts before beginning coding and writing up. The pre-defined codes were used to have a complete picture of the data. NVivo was used to evaluate the qualitative data using a thematic content analysis approach using a theoretical model.

**Ethical consideration:** Ethical clearance was obtained from the Institutional Review Board of Hawassa University, College of Medicine and Health Sciences. An official permission letter from the Regional Health Bureau was secured for Woreda Health offices and health facilities. Official permission was also sought from the selected health facilities. Written informed consent was obtained from participants after confirmation to participate in the study. Personal privacy was maintained by interviewing the interviewee alone and identifications like names were not used in the questionnaire. Participants were also assured that their participation, nonparticipation or their refusal to answer questions had no effect on their personal life.

## Results

**Organizational Governance and Structure:** More than half (54%) of the health centers were located in urban area, and most health posts (89%) were from rural area. Around 14(89%) of primary hospitals were governed by a governing board, and only 50(55%) of health centers were governed by a committee. Out of 92 health centers, only 42 (46%) health center provides inpatient services. Nine (10%) health centers had no isolated labor and delivery room. All health centers and primary hospitals had a 24-hour staffed dedicated emergency unit, and only seven

**Participants Socio-demographic characteristics:** In four regions of Ethiopia, including agrarian and pastoralist areas, we performed a total of 41 KIIs, 32 IDIs, and 13 FGDs. The participants ranged in age from 18 to 69 years old (Table 1). Individual IDIs, KIIs, and FGDs took an average of 30 minutes, whereas FGDs with responsible entities and community leaders took an average of one and a half hours. The following is a summary of the study's findings. (8%) of the health centers have an operating room. Besides, all selected primary hospitals had a 24-hour staffed dedicated emergency unit such as in-patient service, isolated labor and delivery, surgical ward and neonatal intensive care unit (NICU), but only 5 (85.7 %) of hospitals had both medical wards and operation rooms.

**Table 1:** Participant coding, barriers to family planning service utilization, Ethiopia, 2021

code	Region	Role	Age	Sex	Education	Experience, years
001	Oromiya	Midwife	25	F	12+2	5.0
002	Oromiya	HEW	27	F	10+2	3.0
003	Oromiya	Head of the office	27	M	12+4	5.0
004	Oromiya	Pregnant woman	18	F	3	-
005	Oromiya	Youth	19	M	4	-
006	Oromiya	Maternal and child health focal	24	F	12+2	4.0
007	Oromiya	HEW	38	F	10+1	4.0
008	Oromiya	Maternal and child health focal	22	F	12+4	2.5
009	Oromiya	Maternal and child health focal	49	M	12+6	10.0
010	Oromiya	Pregnant woman	23	F	10	-
011	Oromiya	Youth	17	M	11	-
012	Oromiya	Head of health center	24	M	12+2	1.5
013	Oromiya	Head of health post	28	F	10+1	8.0
014	Oromiya	Teacher	20	F	12+2	5.0
015	Oromiya	Mother who gave birth at home	22	F	8	-
016	Oromiya	Youth	15	F	7	-
017	Oromiya	Head of health center	28	M	12+4	5.0
018	Oromiya	HEP coordinator	28	F	12+4	10.0
019	Oromiya	Medical director	33	M	12+6	0.5
020	Oromiya	Head of district health office	31	M	12+4	10.0
021	Oromiya	Woreda MCH health coordinator	42	F	12+6	23.0
022	Oromiya	Reproductive health officer	40	M	12+6	15.0
023	Oromiya	Pregnant woman	35	F	10+2	-
024	Oromiya	Pregnant woman	21	F	4	-

**Essential health service list availability at PHCU and districts:** Before COVID-19 pandemic, 35 (81.4%) of woreda health offices, 67(73%) of the health center, 13(72%) hospitals, and 207 (60%) health posts had a defined list of essential health services, but after COVID-19 pandemic 30 (69.8%) woreda health offices, 58 (63%) of health centers and 229 (66.5%) of health posts received a defined list of EHS (Figure1).

**Barriers to family planning service uptake:** This study identified multifaced barriers of FP which were summarized under four main teams, namely individual, community, health system, and contextual barriers. Under each team, there are 2 to 3 sub-teams that can more easily explain major teams.

### 1. Individual Barrier

#### 1.1. Misconception

The high level of misconception towards family planning was the major barriers towards service utilization and early discontinuation of long-acting family planning. Most of them mentioned that women in their locality believe that long-acting FP like Implanon causes hypotension and predispose them for infectious disease. One of the participants explained that:

*“… Women frequently complain that Implanon decreases their blood pressure and makes them tired. They also believe that they will not be able to stand over a fire for long periods of time to cook. They associate it with Implanon once they insert it, and if they suffer from malaria or other ailments, they associate it with Implanon. As a result, they claim to be able to remove it early. We offer counseling, but we can't deny their claim if they insist on it. Because they complain that the specialists will not be able to take it out when we need it. Others who want to use it will be harmed as a result of this.”*

[27 year old, HEW, KII participant]

Similarly, most FGD and in-depth interview participants believed that using family planning could lead to psychological issues.

*“… women who use the three-month injectable and five-year Implanon become aggressive and “kewes” (crazy)…”*

[A-51-years old TBA, FGD participants]

#### 1.2. Fear of side effects

Fear of contraceptive side effects, such as bleeding, mental illness, infertility, and a change in body image and change in behavior is one of the reasons why study participants do not use FP services. One of the KII participants, for example, said that FP was the cause for infertility.

*“The villagers cite a woman and say “Mother of Mr. X has been using injectable and now unable to give birth therefore they label it infertility. So the community say if you want to give birth again don't take injectable. Which* implying *that it is linked with infertility…”*

[A 31 years old HEW, KII participant]

Additionally, most of the participants mentioned that utilization of family planning alter women's body image and it also increase medical complications.

*“… Particularly the use of Implanon and Depo were considered as the reasons for being fat, because of their hormonal nature. This in turn is believed to result in an increased blood pressure. As a result, it's impacting utilization of FP methods.”*

[31 year old, WDA leader, FGD participant]

#### 1.3. Gender preference

Some participants also reported that gender preference was one of the barriers for FP service utilization.

*“… They often want to have a son so that they do not use family planning until they have a baby boy. Therefore, this reduces the demand of community.”*

[18 year old, pregnant woman, FGD participant]

### 2. Community barrier

#### 2.1. Lack of autonomous decision-making power

The majority of the participants have stressed that women don't have decision making power regarding family planning service utilization. This has been widely mentioned among key informants from agrarian and pastoralist areas. One participant from pastoralist mentioned;

“…. I know one family, who has eight children, and as a result the family is exposed to food shortages. Therefore, a mother has opted to use family planning, but when her husband discovered that his wife was using Implanon, he cut it out with a knife…”

[48 year old, Kebele leader KII participant]

Another key informant from Agrarian reaffirmed that customers couldn't utilize FP due to their husband.

*“… during our counseling, some mothers complain that their husband opposes the idea of using family planning.”*

[42 years old, Woreda Health office RMNCH coordinator, KII participant]

Male dominancy was mentioned as a barrier for FP utilization. Another participant furthered the idea by stating the following.

*“… there is male dominance in our kebele. I heard of a mother who was forced by her husband to get it removed or else he doesn't want to live together with her… But some mothers use the FP by hiding their husbands.”*

[A 28 year old, mother, FGD participant].

#### 2.2. Social norms

Fear of injunctive norm that refers to disapproval of contraceptive practice by friends, parents, and husbands, mother in-law etc., stigmatization, and whisper affect adolescent FP service utilization;

*“… there is fear of community views on using reproductive health services by adolescents. For example, if I go to the health post to take a condom, the community will see me as a wrongdoer and a rude person. In the health post, there is a box for condoms, but no one openly takes the condom as they are afraid of what people say about their behavior”*

[A 35 year old HEW, KII participant,]

#### 2.3. Cultural, traditional beliefs and religious thoughts

In this exploratory study, a range of cultural barriers were found to affect contraceptive use. A participant from the South Omo zone has mentioned how the culture affects the use of contraceptives.

*“As in Bena-Tsemay culture, women are not allowed to use pregnancy prevention methods. They consider using contraceptives dishonorable conduct.”*

[A 40-year-old WDA, IDI participant].

Some other participants also mentioned that the existing social norms do not allow a woman to use FP. The following FGD participant also strengthened this idea.

*“… The community opposes the idea of using pregnancy prevention methods because they believe that women's role is only to bear children, so they should not stop giving birth.”*

[38 year old PHCU head, KII participant]

In some communities, women have long used certain plants to prevent conception, according to a few participants. The community's reliance on traditional contraception may have a direct impact on the adoption of modern family planning technologies.

The majority of individuals indicated religious thoughts as significant factors for low family planning service uptake in the qualitative findings.

*“… Children are insatiable creatures. Jesus Christ will grow and feed the born baby. Therefore, women should give birth.”*

[A-42-years old religious leader, FGD participants]

The community also select some type of contraception as a sinful act. One of the study participants reported that:

*“The community considers use of some types of FP as a sinful act and other types not. They believe IUCD utilization is a sinful act but they accept injectable FP methods, which mainly due to the fact that injectables were introduced earlier than the long acting FP …..”*

[A-30-years old RMCH focal, KII participant]

### 3. Health system barrier

#### 3.1. Structural

##### Shortage of supplies

The main mentioned barrier for FP utilization by almost all study participants is the shortage of supplies. A majority of the participants also reported that there is no demand side barrier.

*“Mothers do not have to contend with a large number of children because one may become ill while another is on the way. For example, a mother might say, “I'm not leaving this room until you give me a contraceptive, please,” but I stress that it's a supply problem, not my unwillingness to give. In our facility, even in our woreda, there is a shortage of injectable and Implanon FP.”*

[A 38 years old MCH focal, KII participant]

Almost all participants in the qualitative study reported that women need to utilize short-acting family planning but due to supply shortage they enforced to use long-acting FP. The below quote best explains these sentences;

*“… The available service does not following client centered approach, which compromises service quality. A woman came for an injectable contraceptive method when it was not available. She was forced to receive implants, and a mother came for implants, but she may receive an injectable method.”*

[A 37-year-old RMNCH regional director, KII participant]

##### Lack of infrastructure and equipment

The participants also mentioned that lack of confidentiality is a barrier for FP service uptake, which is mainly due to a lack of conducive infrastructure and essential.

*“Due to poor infrastructure, we are providing FP and other services in one room … The available HP is not conducive to providing either IUCD or other services. IUCD needs sterilized material, but the HPs didn't have sterilized material, so they couldn't provide it even if they knew.””*

[A 41-year-old, RMNCH regional directorate, KII participant]

#### 3.2. Functional

##### Shortage of skilled manpower

Almost all participants mentioned that there is a shortage of trained health providers in their health facilities and a high turnover of skilled health providers, which has been listed as a FP service utilization barrier.

*“… the other obstacle is the shortage of trained health care providers in our health center. The long-acting FP method was provided by trained professionals, not by everyone. in order to do this, the health center has a shortage of health providers.”*

[A 31 year, PHCU director, KII participant]

Similarly, another KII participant also strengthened that they could not fill the turnover gap due to budget shortage.

*“… The other issue is the shortage of skilled providers and high trained staff turnover and those health facilities that couldn't provide all FP to clients. The Woreda health office is not able to quickly replace or train skilled personnel because of a lack of budget.”*

[A 45 years old, Woreda health office, KII participant]

##### Poor quality of service

Counseling regarding methods of FP and their side effects is crucial for preventing early removal of contraceptives and decreasing frustrations due to some minor side effects. The majority of the participants mentioned that there is a counseling problem on the provider side.

*“The community's attitude towards FP still has a problem because of the poor-quality counseling service provided to them. For instance, early removal of Implanon within 6 months was higher in some areas. This is related to the health care providers' counseling skills and high turnover. While giving FP service, healthcare providers should make clear the FP options available for the mother, rather than prescribe specific methods. If health workers clearly inform the mother of the advantages and possible side effects of each FP method, she may not rush to remove it because of minor problems.”*

[34 years PHCU MCH focal, KII participant]

Similarly, poor-quality service was also another barrier to long-acting contraceptive methods' service utilization. One KII participant bitterly explains that;

*“… As an example, some of the IUCD [Intrauterine Contraceptive Device] service users come to remove it because the swelling was observed, and this problem may indicate poor quality service or an undiagnosed problem of the client during the IUCD insertion.”*

[A 45-year PHCU director, KII participant]

**Poor monitoring and evaluation system:** The presence of strong, consistent, and gap-filling supportive supervision, monitoring, and evaluation is crucial for the improvement and continuity of service utilization. But, the participants of this study stated that there was no strong reporting of performances for higher officials; which makes the service provider negligent. The attention given to the FP service has been declining over the last 2-3 months. This statement was evidenced by the following quote;

*“There was a weekly reporting mechanism, but it is not done. There was an evaluation of maternal health services such as family planning services, but we are not doing it because there is no budget. Previously, the evaluation of maternal services was supported by stakeholders, but in this year there is no one who is interested on it ….”*

[A 34-year-old RMCH officer, KII participant]

Likewise, the monitoring and evaluation system was poor and there was no responsible body for taking the monitoring and evaluation system activity. In most of the health posts, the HEW was not available at the health post for a long period of time to serve the community. The rural community is now facing challenges since there was no other option for them other than the government health facilities like health posts. The health post forgets the service since there is no one who asks the HEWs.

*“… in the last two years it has totally declined and I can say that there was no family planning service in the rural community… The main challenges are that there was no monitoring, evaluation, and also the HEW was not available at the health post for a long period of time… The rural community is now facing challenges since there was no other option for them other than the government health facilities like health posts. They (Health post) forget the service…”*

[A 40-year-old, Quality Team, FGD participant]

### 4. Contextual barrier

#### 4.1. Accessibility

**Lack of access to transportation:** Almost all participants reported that lack of vehicles and access to roads are other barriers that halt strong monitoring and evaluation services.

*“There is a shortage of vehicles for supportive supervision. The providers are not getting any incentive during supportive supervision. There is a shortage of skilled manpower in specific areas of service. Because training was not provided due to the pandemic. The health providers are not well equipped to provide supportive supervision. There are hard to reach areas to give service.”*

[A 30 year old MCH focal, KII participant]

**Hard-to-reach areas:** The participants also reported that hard-to-reach areas with limited access to road and transportation facilities were found to have low uptake of family planning services. The topography and climatic condition of the kebele were also mentioned as factors for family planning service utilization. One of the participants mentioned that:

*“Some of our kebeles (lower administrative units) are remote areas and don't have access to road and transportation facilities that compromise accessibility and utilization of family planning services.”*

[48 year old District RMCH officer, KII participant]

**Conflict/instability:** It is obvious that, in any given setting, armed conflicts can disrupt health service delivery. Disruptions of health service delivery during conflicts exacerbate the risk of unwanted pregnancy due to a shortage of family planning methods in the affected population. Likewise, the family planning service was interrupted for some time due to the current conflict. During the conflict, almost all of the health facilities in the conflict area were closed. One of the participants reported that:

*“… We have started to give the FP services when the hospital was opened as new. But, it was interrupted due to the conflict because this hospital was serving as a military camp.”*

[A 27-year-old RMCH GP, FGD participant]

#### 4.2. COVID-19 pandemic

COVID-19 pandemic has proved to have an impact on essential health service utilization, including family planning services. A majority of the participants agreed that it was very difficult to deliver the family planning service at the early stage of the COVID-19 pandemic. Restriction of movement, all services overwhelmed by COVID-19 related mobilizations, fear of quarantine, no client allowed to enter to facilities without face mask were mentioned as major reasons. One of the participants reported that:

*“In this community, people come to the health center with their families, and this makes it tough for the service. We allowed them to enter the room if they wore a face mask. They were reluctant to wear face masks. The guard at the health center will not allow those so Patients and clients who didn't wear face masks were told to go back home rather than buy a face mask. Thus, the number of women who utilize family planning decreased.”*

[A 30-year-old RMCH focal person, KII participant]

Another KII participant also mentioned that the beginning of the pandemic disrupted FP service provision, but that currently, the problem has been solved.

*“… Our performance at the beginning of the eight months of the COVID pandemic and in the last three months was quite different. In the beginning, everybody was panicked about COVID-19. For example, the FP service was decreased but is showing improvement now.”*

[A 34, years old District health office RMNH coordinator, KII participant]

## Discussion

The study identified individual, community, health-system, and environmental constraints as potential barriers to FP utilization. Individual barriers highlighted by all KII, IDI, and FGD participants include misconceptions about the adverse effects of FP, fear of side effects, and gender preference. Lack of women's autonomy, strong social standards, and cultural and religious views are the key community-side hurdles. The main points cited by almost all survey participants were health care hurdles such as a paucity of FP supply and contextual constraints such as the covid-19 epidemic and topography.

FP can cause irregular bleeding, weight gain, temporary infertility, and other minor side effects, but many women do not even notice them or find that they subside after a few months of use (10). These negative consequences, however, have created a host of myths and misconceptions that have impeded the adoption of FP. In line with this, our research showed that fear of side effects, myths, and misconceptions are the most common individual-based barriers to FP use and that this aggravates early discontinuation of long-acting FP. Many participants believe that using FP causes psychiatric problems, predisposes them to other infections, and increases their chances of being infertile in the future. Poor counseling during FP provision, which is a major problem in our study location, is responsible for these misunderstandings, fears of adverse effects, and misconceptions (11). This finding is also consistent with the findings of other studies(9,12).

An equal decision-making process strengthens the marital relationship and keeps the spouse's physical and mental health(13). However, due to high socio-cultural norms many African countries like Ethiopia far from this and all decision-making power held by themselves, which is a big barrier for FP service utilization(14). Our research also revealed that one of the primary community-level barriers to FP use is a lack of women's autonomy in decision-making. Our research found that if women use FP on their own, their husbands are more likely to physically attack them, and their marriage will end. This could be related to a lack of partner involvement in the FP services, which has a significant impact on boosting partner understanding about FP use and improving various reproductive health issues. This finding is also supported by other studies(14,15).

Social norms, cultural and traditional beliefs, and religious thoughts are mentioned as barriers to FP service uptake by study participants. According to this study, the fear of stigma and being judged by other members of the community, this was the primary reason that teenagers did not use FP services (16). Due to their religion, participants considered the use of FP as a sinful act, and children are gifts from God. This might be because most religions do not encourage taking FP service in general and strong and continuous health education can alleviate such a problem(17).

According to the Donabedian framework, quality of care can be measured by structure, process, and outcome. These three components are intertwined with each other; that means improved structure leads to improved processes, which leads to improved outcomes (18). In agreement with this, the main barriers in our study area include poor structures such as unavailability of trained personnel, lack of privacy due to poor infrastructure status, and paucity of supplies, all of which have an impact on the FP service process and outcome. It is suggested that providing FP in a private room increases provider-client interaction, increases counseling of side effects, and client satisfaction, which prevents unnecessary misconceptions, in-increase service uptake, and has a long-term impact on lowering fertility rates and maternal and child morbidity and mortality. The finding is supported by other studies (19,20).

Conducting practical training with strong mentorship and supervision has a great effect on FP service provision and uptake. This is very helpful, especially for rural health facilities like health posts, because health extension workers have limited capacity, especially on long-acting family planning provisions. Therefore, through mentorship, it is easy to create skilled healthcare providers (21). However, our study explored that there is poor M&E due to a lack of budget, a lack of skilled manpower, a shortage of transportation, and topography. Due to these barriers, the health center to HP and HP to community linkage were weakened.

The covid-19 pandemic turns the attention of routine services to and causes restrictions on movement and fear of quarantine, which creates fear of visiting health facilities and shortages of supplies due to lockdown. Due to this covid-19 disrupted FP uptake and also other routine services (22). Similarly, our study explores Covid-19 was one of the barriers to utilizing FP services, and this study is also supported by another study done in Nairobi (23).

This study also identified that conflict influenced FP service uptake. Disruptions of health service delivery during conflicts exacerbate the risk of unwanted pregnancy due to a shortage of family planning methods in the affected population. Likewise, the family planning service was interrupted for some time due to the current conflict.

The study identified a range of barriers towards utilization of FP in four main teams, namely individual, community, health care, and contextual barriers. Misconceptions about the side effects of FP, fear of side effects, and gender preference were the main points that were mentioned by all KII, IDI, and FGD participants as individual barriers. Lack of women's autonomy, strong social norms that stigmatize adolescents and affect FP utilization, and cultural and religious thought are the main community side barriers. Healthcare barriers like shortage of FP supply, poor M&E system, poor health providers' capacity, and contextual barriers such as covid-19 pandemic and topography were the main points that were raised by almost all study participants. As a result, robust and consistent health education to lessen the impact of sociocultural norms, misconceptions, and religious obstacles, as well as partner engagement, reduces misconceptions and women's autonomy. It is critical to increase FP counseling by offering training for health care providers, as well as strengthening mentorship and supervision programs in collaboration with other governmental and non-governmental organizations, in order to develop cost-effectively skilled health providers. It's also crucial to have all of the necessary materials on hand in order to enhance service take-up.
